# Effect of Annealing Temperature on Electrochemical Properties of Zr_56_Cu_19_Ni_11_Al_9_Nb_5_ in PBS Solution

**DOI:** 10.3390/ma16093389

**Published:** 2023-04-26

**Authors:** Zhiying Zhang, Xinwei Zhong, Xiujin Teng, Yanshu Huang, Han Han, Tao Chen, Qinyi Zhang, Xiao Yang, Yanlong Gong

**Affiliations:** 1 School of Materials Science and Engineering, Wuhan University of Technology, Wuhan 430070, China; zhongxinwei2002@whut.edu.cn (X.Z.); txj2023@whut.edu.cn (X.T.); 308201@whut.edu.cn (Y.H.); hanhan12138@whut.edu.cn (H.H.); zhqy@whut.edu.cn (Q.Z.); 317630@whut.edu.cn (X.Y.); gongyanlong2000@163.com (Y.G.); 2 Yang Jiang Alloy Laboratory, Yangjiang 529568, China; 3 Xiangyang City Liqiang Mechanics Limited Company, Xiangyang 441799, China; hjhm666@163.com

**Keywords:** metallic glass, annealing, phosphate buffer saline (PBS) solution, corrosion, electrochemical properties

## Abstract

The electrochemical properties of as-cast Zr_56_Cu_19_Ni_11_Al_9_Nb_5_ metallic glass and samples annealed at different temperatures were investigated using potentiodynamic polarization tests and electrochemical impedance spectroscopy (EIS) in phosphate buffer saline (PBS) solution. It was shown that passivation occurred for the as-cast sample and the samples annealed at 623–823 K, indicating good corrosion resistance. At higher annealing temperature, the corrosion resistance first increased, and then decreased. The sample annealed at 823 K exhibited the best corrosion resistance, with high spontaneous corrosion potential E_corr_ at −0.045 V_SCE_, small corrosion current density i_corr_ at 1.549 × 10^−5^ A·cm^−2^, high pitting potential E_pit_ at 0.165 V_SCE_, the largest arc radius, and the largest sum of R_f_ and R_ct_ at 5909 Ω·cm^2^. For the sample annealed at 923 K, passivation did not occur, with low E_corr_ at −0.075 V_SCE_, large i_corr_ at 1.879 × 10^−5^ A·cm^−2^, the smallest arc radius, and the smallest sum of R_f_ and R_ct_ at 2173 Ω·cm^2^, which suggested the worst corrosion resistance. Proper annealing temperature led to improved corrosion resistance due to structural relaxation and better stability of the passivation film, however, if the annealing temperature was too high, the corrosion resistance deteriorated due to the chemical inhomogeneity between the crystals and the amorphous matrix. Optical microscopy and scanning electron microscopy (SEM) examinations indicated that localized corrosion occurred. Results of energy dispersive X-ray spectroscopy (EDS) and X-ray photoelectron spectroscopy (XPS) illustrated that the main corrosion products were ZrO_2_, CuO, Cu_2_O, Ni(OH)_2_, Al_2_O_3_, and Nb_2_O_5_.

## 1. Introduction

Zr-based bulk metallic glasses (BMGs) have attracted intense concern due to their high strength, high hardness, large elastic limit, good corrosion resistance, good glass-forming ability (GFA), large critical size, and good biocompatibility [1,2,3,4,5,6]. Corrosion resistance was affected by the chemical composition, microstructure, heat treatment conditions, the types and concentrations of corrosive media, temperature, and so on. With higher Nb content, the corrosion resistance of Zr_46_Cu_30.14−x_Nb_x_Ag_8.36_Al_8_Be_7.5_ (x = 0, 2, 5, 10) in 0.1 mol/L HCl, 0.5 mol/L NaCl, and 0.5 mol/L H_2_SO_4_ solutions was improved due to the promoted oxidation of Zr and thicker oxide film [7]. The electrochemical properties of Zr_56.2_Ti_13.8_Nb_5.0_Cu_6.9_Ni_5.6_Be_12.5_ composite with crystalline dendrites in an amorphous matrix in 1 mol/L NaCl solution showed that corrosion in the amorphous matrix was faster [8]. In 1 mol/L HCl solution, the corrosion resistance is as follows: Zr_55_Ti_4_Y_1_Al_10_Cu_20_Ni_7_Co_2_Fe_1_ > Zr_50_Ti_4_Y_1_Al_10_Cu_25_Ni_7_Co_2_Fe_1_ > Zr_55_Ti_4_Y_1_Al_12_Cu_18_Ni_7_Co_2_Fe_1_ > Zr_60_Al_12_Cu_28_ > Zr_60_Al_10_Cu_30_ > Zr_55_Al_10_Cu_35_ [9]. Zr_55_Ti_4_Y_1_Al_10_Cu_20_Ni_7_Co_2_Fe_1_ exhibited the highest corrosion potential E_corr_ at −0.420 V_SCE_, and the smallest corrosion current density i_corr_ at 1.0 × 10^−7^ A·cm^−2^, suggesting the best corrosion resistance. Zr_55_Al_10_Cu_35_ illustrated the lowest E_corr_ at −1.165 V_SCE_, and the largest i_corr_ at 3.0 × 10^−7^ A·cm^−2^, suggesting the worst corrosion resistance. The corrosion resistance is better with higher E_corr_ and smaller i_corr_. Zr_55_Ti_4_Y_1_Al_10_Cu_20_Ni_7_Co_2_Fe_1_, Zr_50_Ti_4_Y_1_Al_10_Cu_25_Ni_7_Co_2_Fe_1_ and Zr_55_Ti_4_Y_1_Al_12_Cu_18_Ni_7_Co_2_Fe_1_ showed higher content of Ti (4 at.%), Ni (7 at.%), and Co (2 at.%) and lower content of Cu (18–25 at.%) than Zr_60_Al_12_Cu_28_, Zr_60_Al_10_Cu_30_, and Zr_55_Al_10_Cu_35_, with better corrosion resistance. Cu decreased the corrosion resistance due to the deterioration of the protection of the film, and Ti, Ni, and Co increased the corrosion resistance because of the enhancement of the protection of the film. The electrochemical properties of Zr_52_Al_10_Ni_6_Cu_32_ in 0.05–0.5 mol/L NaCl and 0.05–0.5 mol/L NaF solutions indicated that corrosion resistance decreased with higher concentrations of Cl^−^ and F^−^ [10]. Zr_60_Fe_10_Cu_20_Al_10_ samples were prepared using selective laser melting with a laser power of 200 W and an exposure time of 40–70 μs, and their corrosion resistance in 3.5 wt.% NaCl solution decreased with the increase in exposure time [11].

Zr-based BMGs showed promising biomedical applications, such as orthopedic and dental device materials [12]. Zr_55.8_Al_19.4_Co_17.36_Cu_7.44_ exhibited good corrosion resistance in phosphate-buffered saline (PBS) solution, combined with good glass-forming ability, a large critical diameter of 12 mm, a high yield strength of 2 GPa, and a high fracture toughness of 120 MPa·m^1/2^ [13]. Zr_60.5_Hf_3_Al_9_Fe_4.5_Cu_23_ showed good corrosion resistance in PBS solution, a large critical diameter of 10 mm, a high yield strength of 1.64 GPa, a large plastic strain of 4.0%, and good biocompatibility and wear resistance [14]. Zr_58.6_Al_15.4_Co_18.2_Cu_7.8_ illustrated good corrosion resistance in PBS solution, a large critical diameter of 10 mm, a high yield strength of 1.95 GPa, a large plastic strain of 2.0%, and good antibacterial properties [15]. Zr_65−x_Ti_x_Cu_20_Al_10_Fe_5_ (x = 0–8) exhibited better corrosion resistance with higher Ti content, and the mechanical properties were the best with 2% Ti [16]. The corrosion resistance of Zr_53_Al_16_Co_26_M_5_ (M = Pd, Au and Pt) in PBS solution was the best for Pt due to the increased Zr content and decreased Al content in the passive film, and the worst for Au [17]. Zr_45_Ti_36_Fe_11_Al_8_ showed better corrosion resistance than commercially pure Ti in PBS solution [18]. Zr_55_Cu_30_Ni_5_Al_10_ exhibited poorer corrosion resistance than the medical grade ASTM F 75 cast CoCrMo alloy and AISI 316LVM low carbon vacuum re-melted stainless steel alloy in PBS solution at a body temperature of 310 K [19]. Zr_40_Ti_37_Co_12_Ni_11_, Zr_50_Ti_32_Cu_13_Ag_5_, Zr_46_Ti_40_Ag_14_, and Zr_46_Ti_43_Al_11_ indicated better corrosion resistance than commercially pure Ti and 316L stainless steel in PBS solution [20,21].

The mechanical properties and the corrosion and electrochemical properties of Zr-based BMGs were affected by annealing conditions, such as annealing temperature and annealing time. Proper annealing led to the formation of nanocrystals in the amorphous matrix, which acted as the initiation sites for shear bands and hindered the propagation of shear bands. Therefore, the shear band density was increased, resulting in improved plasticity [22]. If the annealing temperature was too high or the annealing time was too long, large size crystals were formed in the amorphous matrix, and strength and plasticity decreased due to the stress concentration and formation of microcracks at the interfaces. Zr_65_Cu_17.5_Fe_10_Al_7.5_ samples were annealed at 573 K, below the glass transition temperature T_g_, for 0.5–4 h, and a large plasticity of 7.1%, high hardness of 487 HV, and improved pitting corrosion resistance in 3.5% NaCl solution was obtained for the sample annealed for 1 h, due to the formation of nanocrystals in the amorphous matrix, the reduced free volume, and the increased shear band density [22]. With the increase in annealing time, the plasticity, the hardness, the pit potential E_pit_, and the passivation region E_pit_–E_corr_ first increased, and then decreased. Zr_58_Nb_3_Cu_16_Ni_13_Al_10_ samples were annealed at 523 K, 673 K (lower than T_g_), 773 K (higher than the crystallization temperature T_x_), and 873 K for 6 h, and at the higher annealing temperature, the corrosion resistance in 1 mol/L H_2_SO_4_ solution at 333 K deteriorated [23]. Zr_60_Cu_20_Ni_8_Al_7_Hf_3_Ti_2_ samples were annealed below T_g_, and the good corrosion resistance in H_2_SO_4_ solution was maintained [24]. Zr_50.7_Ni_28_Cu_9_Al_12.3_ samples were annealed at 719 K (T_g_~T_x_), 768 K (>T_x_), and 810 K for 30 min, and the electrochemical properties in 0.5 mol/L H_2_SO_4_, 1 mol/L NaCl, and 1 mol/L HCl solutions showed that the corrosion resistance was the best for the sample annealed at 768 K due to the proper quantity of ZrO_2_ nanocrystals in the amorphous matrix [25]. Zr_41.2_Cu_12.5_Ni_10_Ti_13.8_Be_22.5_ and Zr_57_Cu_15.4_Ni_12.6_Al_10_Nb_5_ samples were annealed at 0.9 T_g_ for 4 h, and the corrosion resistance in NaCl solution was improved due to the reduced free volume [26]. Zr_68_Al_8_Ni_8_Cu_16_ samples were annealed at 673 K and 713 K with a crystallinity of 10% and 77%, and the corrosion resistance in 1 mol/L HCl solution decreased at the higher annealing temperature [27]. Zr_59_Ti_6_Cu_17.5_Fe_10_Al_7.5_ samples were annealed at 573 K (<T_g_) for 0.5–4 h, and the corrosion resistance remained good in PBS solution [28].

The effects of annealing temperature and time on the corrosion and electrochemical properties of Zr-based BMGs and the composites in simulated body fluids, such as PBS solution, were rarely reported, and systematic study was needed. In this work, the corrosion and electrochemical properties of the as-cast Zr_56_Cu_19_Ni_11_Al_9_Nb_5_ metallic glass and the samples annealed at different temperatures (<T_g_, T_g_–T_x_, >T_x_) in PBS solution were investigated using potentiodynamic polarization tests, electrochemical impedance spectroscopy (EIS), optical microscopy, scanning electron microscopy (SEM), energy dispersive X-ray spectroscopy (EDS), and X-ray photoelectron spectroscopy (XPS). Our findings make contributions to the research and development of Zr-based metallic glass and composites in biomedical applications.

## 2. Materials and Methods

### 2.1. Material Preparation

Zr_56_Cu_19_Ni_11_Al_9_Nb_5_ metallic glass was prepared using copper-mold suction after the arc-melting of Zr, Cu, Ni, Al, and Nb with high purity under vacuum conditions and filled with Ar. Remelting was carried out 5 times to obtain chemical homogeneity. The samples were cut to a size around 5 mm × 4 mm × 1 mm using a SYJ-160 low speed diamond saw (Shenyang Kejing Automatic Equipment Limited Company, Shenyang, China). The samples were annealed at 623 K (below T_g_), 723 K (between T_g_ and T_x_), 823 K (above T_x_), and 923 K (far above T_x_) for 30 min, and then cooled to room temperature. The sample surfaces were ground using 800, 1200, and 1500 grit sandpaper, polished using 2.5 and 0.5 μm diamond paste, and then cleaned in acetone.

### 2.2. Tests

Electrochemical tests of the as-cast samples and the annealed samples in PBS solution were performed using a CHI660E electrochemical station (Shanghai CH Instruments, Shanghai, China). Saturated calomel electrode (SCE) was the reference electrode, and the graphite electrode was the counter electrode. The sample was the working electrode. The PBS solution contained 8.0 g/L NaCl, 1.44 g/L Na_2_HPO_4_, 0.24 g/L KH_2_PO_4_, and 0.20 g/L KCl, and it was prepared using reagent-grade chemicals and deionized water. The electrochemical tests were performed in the open air at room temperature. In potentiodynamic polarization tests and EIS tests, the electrodes were first stabilized in PBS solution at open circuit potential (OCP) for 60 min. In potentiodynamic polarization tests, the potential was scanned from −0.8 V_SCE_ to 0.8 V_SCE_ at the rate of 0.33 mV/s, and auto-sensitivity was set. In EIS tests, alternative current impedance mode was chosen, and the initial potential was set at the OCP. The frequency was in the range of 10^−2^–10^5^ Hz. The amplitude was 5 mV, and the stabilization period was 2 s. The complex impedance was measured. Zsimpwin software was used to fit the EIS data using the proper equivalent circuit. SH11/YF-III optical microscopy, Zeiss Ultra Plus field emission scanning electron microscopy, X-Max 50× energy dispersive X-ray spectroscopy, and Thermo Scientific ESCALAB 250Xi X-ray photoelectron spectroscopy were used to study the corrosion morphology and corrosion products after the potentiodynamic polarization tests.

## 3. Results and Discussion

### 3.1. Electrochemical Tests

**Figure 1:**
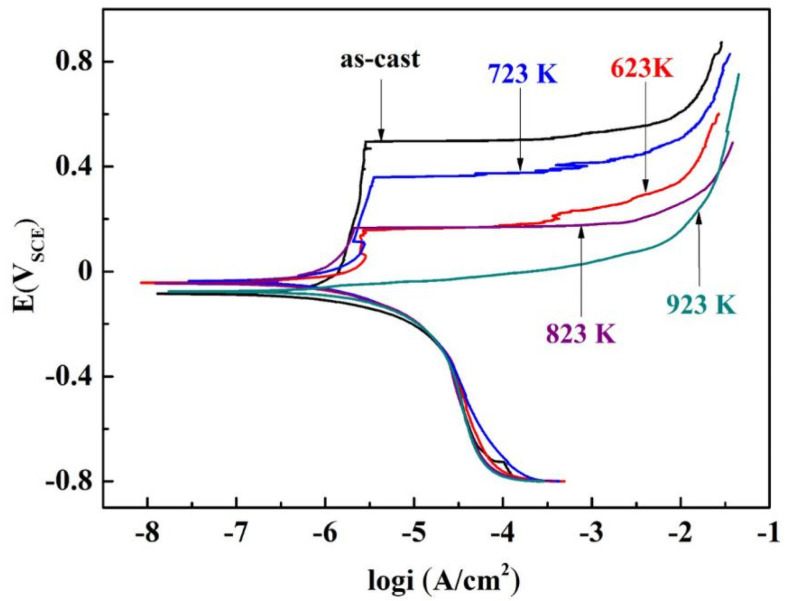
The potentiodynamic polarization curves for the as-cast Zr_56_Cu_19_Ni_11_Al_9_Nb_5_ sample and the samples annealed at 623–923 K in PBS solution.

Figure 1 shows the potentiodynamic polarization curves for the as-cast Zr_56_Cu_19_Ni_11_Al_9_Nb_5_ sample and the samples annealed at 623–923 K in PBS solution, and Table 1 summarizes the obtained electrochemical parameters. Similar trends were observed for the as-cast sample and the samples annealed at 623–823 K, and passivation occurred. With the increase in annealing temperature, the spontaneous corrosion potential E_corr_ gradually increased and then decreased, and the corrosion current density i_corr_ gradually decreased and then increased. Corrosion resistance increased with higher E_corr_, higher pitting potential E_pit_, smaller i_corr_, and a larger passivation region E_pit_–E_corr_. For the as-cast sample, E_corr_ was the lowest at −0.083 V_SCE_, the pitting potential E_pit_ was the highest at 0.496 V_SCE_, and the width of passivation region E_pit_–E_corr_ was the largest at 0.579 V. Passivation occurred for the as-cast sample and the samples annealed at 623 K (below T_g_), 723 K (between T_g_ and T_x_), and 823 K (above T_x_), indicating good corrosion resistance. For the sample annealed at 623 K, E_corr_ was higher at −0.042 V_SCE_, i_corr_ was smaller at 1.466 × 10^−5^ A·cm^−2^, E_pit_ was lower at 0.157 V, and the passivation region E_pit_–E_corr_ was smaller at 0.199 V, suggesting similar corrosion resistance. For the sample annealed at 723 K, E_corr_ was the highest at −0.036 V_SCE_, and i_corr_ was the smallest at 9.977 × 10^−6^ A·cm^−2^, with a wide passivation region E_pit_–E_corr_ of 0.395 V, indicating excellent corrosion resistance. The sample annealed at 823 K exhibited high E_corr_ at −0.045 V_SCE_, small i_corr_ at 1.549 × 10^−5^ A·cm^−2^, high E_pit_ at 0.165 V_SCE_, and a wide passivation region E_pit_–E_corr_ of 0.210 V, indicating good corrosion resistance. For the sample annealed at 923 K, i_corr_ was the largest at 1.879 × 10^−5^ A·cm^−2^, and passivation did not occur, indicating the worst corrosion resistance in PBS solution. With the increase in annealing temperature, the corrosion resistance gradually increased and then decreased.

**Figure 2:**
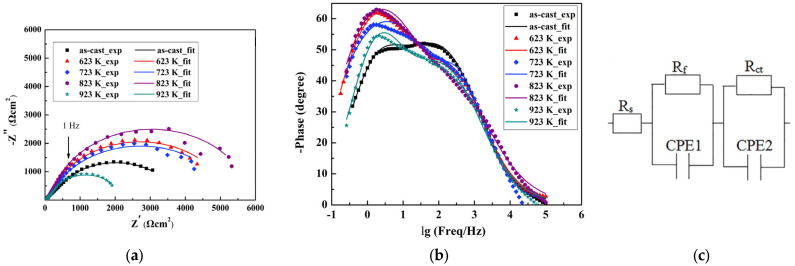
The EIS results for the as-cast Zr_56_Cu_19_Ni_11_Al_9_Nb_5_ sample and the samples annealed at 623–923 K in PBS solution. (**a**) Nyquist plots, (**b**) Bode plots, and (**c**) the equivalent circuit diagram.

Figure 2 illustrates the Nyquist plots, Bode plots, and the equivalent circuit diagram for the EIS results of the as-cast Zr_56_Cu_19_Ni_11_Al_9_Nb_5_ sample and the samples annealed at 623–923 K in PBS solution, and Table 2 shows the fitting parameters. The Nyquist plots exhibit half-circles, which suggest that the control step is the electrochemical reaction accompanied by the transfer of electrons. The Bode plots illustrate two time-constants, with frequencies around 3 Hz and 100 Hz. The maximum phase is reached around 3 Hz. The equivalent circuit diagram as shown in Figure 2c was previously used for the fitting of EIS results of Ni_70_Cr_21_Si_0.5_B_0.5_P_8_ and Ni_72.65_Cr_7.3_Si_6.7_B_2.15_Fe_8.2_Mo_3_ glassy alloys in 1–12 mol/L HNO_3_ solution [29], and EIS results of Zr_65_Cu_17.5_Al_7.5_Ni_10−x_Co_x_ in 3.5% NaCl solution [30]. The passivation film consists of two layers, i.e., the compact inner layer and the porous outer layer. R_s_ is the solution resistance between the working electrode and the reference electrode. R_f_ is the film resistance, and R_ct_ is the charge transfer resistance at the interface between the solution and the film. The constant phase element (CPE) represents the capacitance, considering the surface roughness and inhomogeneity. CPE1 and CPE2 are the constant phase elements for the inner layer and outer layer of the passivation film. The CPE impedance is $Z_{CPE}={Y_{0}}^{-1}\omega^{-n}\left[\cos\left(\frac{n\pi }{2}\right)-j\;\sin\left(\frac{n\pi }{2}\right)\right]$. Y_0_ is the constant, ω is the angular frequency, and n is the parameter between 0 and 1. The sample annealed at 823 K illustrates the largest arc radius and the largest sum of R_f_ and R_ct_, 5909 Ω·cm^2^, indicating the best corrosion resistance in PBS solution. The sample annealed at 923 K illustrates the smallest arc radius and the smallest sum of R_f_ and R_ct_, 2173 Ω·cm^2^, indicating the worst corrosion resistance in PBS solution. With the increasing annealing temperature, the corrosion resistance first increases, and then decreases, which is in agreement with the potentiodynamic polarization results.

### 3.2. Optical Microscopy Observation

**Figure 3:**
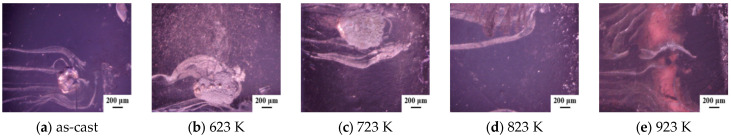
The optical microscopy images of the as-cast Zr_56_Cu_19_Ni_11_Al_9_Nb_5_ sample and the samples annealed at 623–923 K after potentiodynamic polarization tests in PBS solution.

Figure 3 shows the optical microscopy images of the as-cast Zr_56_Cu_19_Ni_11_Al_9_Nb_5_ sample and the samples annealed at 623–923 K after potentiodynamic polarization tests in PBS solution. Obvious localized corrosion occurred for the as-cast sample and the samples annealed at 623 K and 723 K. Minor corrosion was observed in the sample annealed at 923 K. Corrosion was not obvious for the sample annealed at 823 K, suggesting good corrosion resistance.

### 3.3. SEM and EDS Analysis

**Figure 4:**
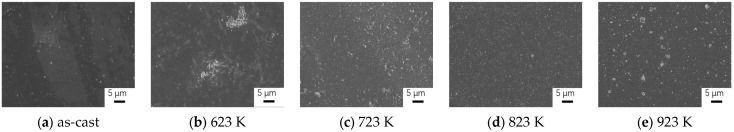
SEM images of the as-cast Zr_56_Cu_19_Ni_11_Al_9_Nb_5_ sample and the samples annealed at 623–923 K after potentiodynamic polarization tests in PBS solution.

**Figure 5:**
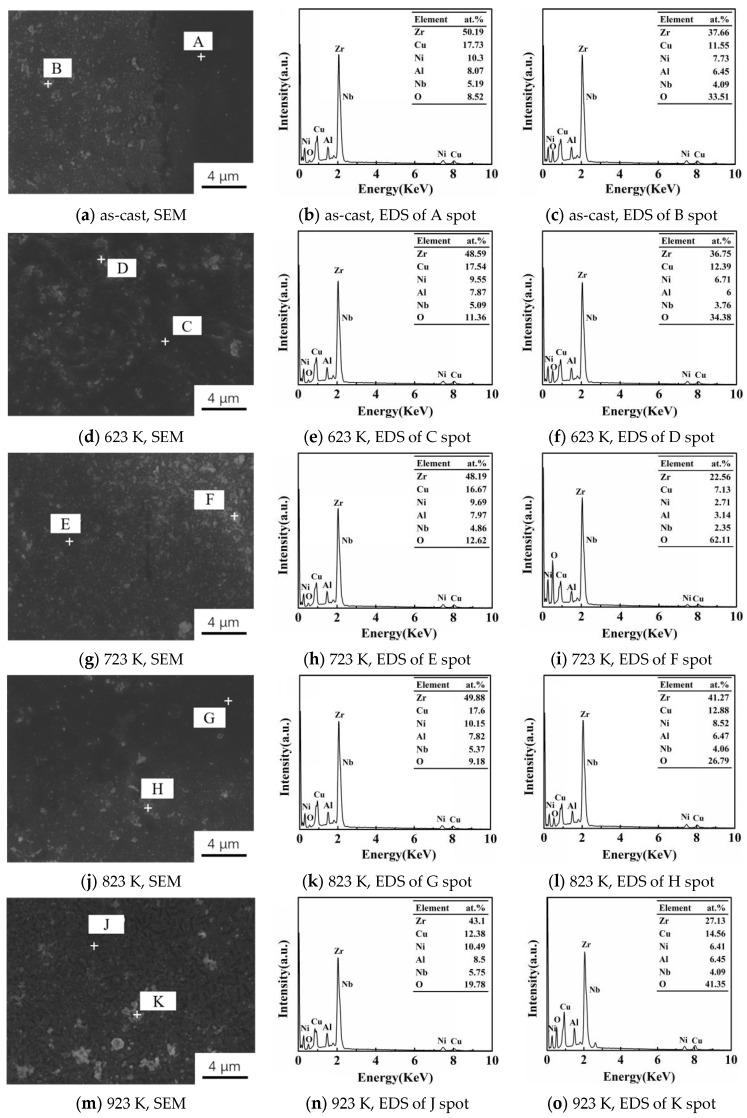
SEM and EDS results of the as-cast Zr_56_Cu_19_Ni_11_Al_9_Nb_5_ sample and the samples annealed at 623–923 K after potentiodynamic polarization tests in PBS solution. Spots A, C, E, G, and J are in the smooth non-corroded area, and spots B, D, F, H, and K are in the corroded area.

SEM images and EDS analysis of the as-cast Zr_56_Cu_19_Ni_11_Al_9_Nb_5_ sample and the samples annealed at 623–923 K after potentiodynamic polarization tests in PBS solution are shown in Figure 4 and Figure 5. Localized corrosion occurred for all the samples. The weakest corrosion was observed in the sample annealed at 823 K, indicating the best corrosion resistance. Spots A, C, E, G, and J are in the smooth non-corroded area, in which the oxygen content was low, less than 20%, and the content of Zr, Cu, Ni, Al, and Nb was close to the original content. On the other hand, spots B, D, F, H, and K are in the corroded area, in which the oxygen content was higher, and the content of Zr, Cu, Ni, Al, and Nb was lower than the original content.

While annealing below T_g_, structural relaxation led to reduced free volume, resulting in improved corrosion resistance [22,26]. When the annealing temperature was slightly above T_x_, crystallization occurred, leading to the formation of nanocrystals in the amorphous matrix. The stability of the passivation film was increased, resulting in better corrosion resistance. When the annealing temperature was well above T_x_, the size of the crystals increased. Corrosion was susceptible to occurring at the interface between the crystals and amorphous matrix due to the chemical inhomogeneity, leading to reduced corrosion resistance.

### 3.4. XPS Analysis

**Figure 6:**
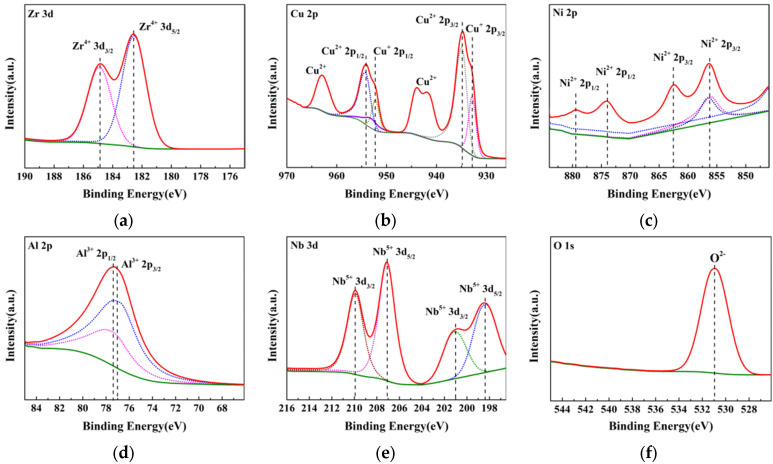
XPS analysis of the Zr_56_Cu_19_Ni_11_Al_9_Nb_5_ sample annealed at 723 K after potentiodynamic polarization test in PBS solution, (**a**) Zr 3d, (**b**) Cu 2p, (**c**) Ni 2p, (**d**) Al 2p, (**e**) Nb 3d, and (**f**) O 1s.

Figure 6 shows the XPS analysis of the Zr_56_Cu_19_Ni_11_Al_9_Nb_5_ sample annealed at 723 K after the potentiodynamic polarization test in PBS solution. In the spectra, the peaks with binding energy of 182.58 eV and 184.88 eV represented Zr^4+^ 3d_5/2_ and Zr^4+^ 3d_3/2_. The peaks at 932.91 eV and 934.33 eV indicated Cu^+^ 2p_3/2_ and Cu^2+^ 2p_3/2_, and the peaks at 952.24 eV and 954.19 eV corresponded to Cu^+^ 2p_1/2_ and Cu^2+^ 2p_1/2_. The peaks at 856.29 eV and 862.38 eV suggested Ni^2+^ 2p_3/2_, and the peaks at 874.12 eV and 879.38 eV indicated Ni^2+^ 2p_1/2_. The peaks at 77.17 eV and 77.32 eV represented Al^3+^ 2p_3/2_ and Al^3+^ 2p_1/2_. The peaks at 198.47 eV and 207.11eV indicated Nb^5+^ 3d_5/2_, and the peaks at 200.93 eV and 209.92 eV represented Nb^5+^ 3d_3/2_. The peak at 530.95 eV suggested O^2−^. The corrosion products mainly consist of ZrO_2_, CuO, Cu_2_O, Ni(OH)_2_, Al_2_O_3_, and Nb_2_O_5_.

Relative to the potential of a standard hydrogen electrode (SHE), the electrode potential of Al/Al^3+^, Zr/Zr^4+^, Nb/Nb^5+^, Ni/Ni^2+^, Cu/Cu^2+^, and Cu/Cu^+^ is −1.662 V_SHE_, −1.529 V_SHE_, −1.200 V_SHE_, −0.250 V_SHE_, 0.337 V_VSH_, and 0.521 V_SHE_. At lower potential, the metal element is more active and is easier to corrode. Al, Zr, and Nb are more active than Ni and Cu, and they are corroded first with the corrosion products of Al_2_O_3_, ZrO_2_, and Nb_2_O_5_. Ni and Cu are then corroded with the corrosion products of Ni(OH)_2_, Cu_2_O, and CuO. The corrosion products of the Zr_56_Cu_19_Ni_11_Al_9_Nb_5_ sample are mainly ZrO_2_, CuO, Cu_2_O, Ni(OH)_2_, Al_2_O_3_, and Nb_2_O_5_. Passivation occurred due to the formation of oxide film or the adsorption of oxygen atoms or oxygen ions. The Cl^−^ ions in PBS solution, which contained 8.0 g/L NaCl, 0.20 g/L KCl, 1.44 g/L Na_2_HPO_4_ and 0.24 g/L KH_2_PO_4_, caused the localized corrosion of the as-cast and annealed Zr_56_Cu_19_Ni_11_Al_9_Nb_5_ samples. According to the oxide-film theory, the Cl^−^ ions caused the localized dissolution of the passivation film, leading to localized corrosion. According to the competitive adsorption theory, the localized preferential adsorption of Cl^−^ ions hindered the adsorption of oxygen atoms or oxygen ions, resulting in the localized corrosion.

## 4. Conclusions

Passivation occurred for the as-cast Zr_56_Cu_19_Ni_11_Al_9_Nb_5_ metallic glass and the samples annealed at 623 K (below T_g_), 723 K (between T_g_ and T_x_), and 823 K (above T_x_), indicating good corrosion resistance in PBS solution. Passivation did not occur for the sample annealed at 923 K (far above T_x_). With the increase in annealing temperature, the corrosion resistance first increased, and then decreased. The sample annealed at 823 K exhibited high E_corr_ at −0.045 V_SCE_, small i_corr_ at 1.549 × 10^−5^ A·cm^−2^, high E_pit_ at 0.165 V_SCE_, a wide passivation region E_pit_–E_corr_ of 0.210 V, the largest arc radius, and the largest sum of R_f_ and R_ct_, 5909 Ω·cm^2^, indicating the best corrosion resistance in PBS solution. For the sample annealed at 923 K, passivation did not occur, and the sample illustrated the highest i_corr_ at 1.879 × 10^−5^ A·cm^−2^, the smallest arc radius, and the smallest sum of R_f_ and R_ct_, 2173 Ω·cm^2^, indicating the worst corrosion resistance in PBS solution.

Optical microscopy, SEM, EDS, and XPS analysis showed that localized corrosion occurred for the as-cast Zr_56_Cu_19_Ni_11_Al_9_Nb_5_ sample and the samples annealed at 623–923 K. In the non-corroded area, the content of Zr, Cu, Ni, Al, and Nb was close to the original content of the sample. In the corroded area, the content of Zr, Cu, Ni, Al, and Nb was lower than the original content. The main corrosion products are ZrO_2_, CuO, Cu_2_O, Ni(OH)_2_, Al_2_O_3_, and Nb_2_O_5_.

The proper annealing temperature led to improved corrosion resistance. When the annealing temperature was below T_g_, structural relaxation led to reduced free volume, resulting in improved corrosion resistance. When the annealing temperature was slightly above T_x_, crystallization started to occur, and the formation of nanocrystals in the amorphous matrix led to improved stability of the passivation film, resulting in better corrosion resistance. However, if the annealing temperature was well above T_x_, the size of the crystals increased, and the chemical inhomogeneity led to corrosion at the interface between the crystals and amorphous matrix, resulting in reduced corrosion resistance. Zr_56_Cu_19_Ni_11_Al_9_Nb_5_ metallic glass and the samples annealed at the proper temperature are promising candidate materials for biomedical applications.

## Figures and Tables

**Table 1:** The electrochemical parameters obtained from the potentiodynamic polarization curves for the as-cast and annealed Zr_56_Cu_19_Ni_11_Al_9_Nb_5_ samples in PBS solution.

Annealing Temperature (K)	E_corr_ (V_SCE_)	i_corr_ (A/cm^2^)	E_pit_ (V_SCE_)	E_pit_–E_corr_ (V)
As-cast	−0.083	1.866 × 10^−5^	0.496	0.579
623	−0.042	1.466 × 10^−5^	0.157	0.199
723	−0.036	9.977 × 10^−6^	0.359	0.395
823	−0.045	1.549 × 10^−5^	0.165	0.210
923	−0.075	1.879 × 10^−5^	/	/

**Table 2:** The EIS fitting results for the as-cast Zr_56_Cu_19_Ni_11_Al_9_Nb_5_ sample and the samples annealed at 623–923 K in PBS solution.

Annealing Temperature (K)	EIS Fitting Results							
	R_s_ (Ω·cm^2^)	Y_01_ (Ω^−1^·cm^−2^·s^n^)	n_1_	R_f_ (Ω·cm^2^)	Y_02_ (Ω^−1^·cm^−2^·s^n^)	n_2_	R_ct_ (Ω·cm^2^)	R_ct_ + R_f_ (Ω·cm^2^)
As-cast	18.1	1.25 × 10^−4^	0.792	3647	1.88 × 10^−4^	0.696	204	3851
623	11.6	1.56 × 10^−4^	0.846	5224	2.71 × 10^−4^	0.675	92	5316
723	13.0	1.64 × 10^−3^	0.793	5303	1.50 × 10^−4^	0.775	57	5360
823	11.5	4.50 × 10^−4^	0.562	225	1.30 × 10^−4^	0.913	5684	5909
923	12.0	1.50 × 10^−4^	0.914	2010	2.77 × 10^−4^	0.659	163	2173

## Data Availability

The data presented in this study are available on request from the corresponding authors. The data are not publicly available due to privacy reasons.
